# Temporomandibular joint disorders in dental students - a systematic review of the literature

**DOI:** 10.1186/s12903-026-08719-3

**Published:** 2026-05-30

**Authors:** Natalia Walczuk, Adrian Borawski, Piotr Skomro, Małgorzata Wójcik, Helena Gronwald, Magdalena Sroczyk-Jaszczyńska, Diana Masłyk, Izabela Barczyk, Marta Grzegocka, Karina Kijak, Adam Wiejkuć, Lidia Szczucka, Adam Andrzej Garstka, Jakub Kozłowski, Danuta Lietz-Kijak

**Affiliations:** 1 Department of Propaedeutic, Physical Diagnostics and Dental Physiotherapy, Faculty of Medicine and Dentistry, Pomeranian Medical University in Szczecin, Szczecin, 70-204 Poland; 2 Department of Physiotherapy, Faculty of Sport Sciences in Gorzów Wlkp, Poznan University of Physical Education, Poznan, 61-871 Poland; 3 Department of General, Dental and Interventional Radiology, Faculty of Medicine and Dentistry, Pomeranian Medical University in Szczecin, Szczecin, 70-204 Poland; 4 Hubala Dental Clinic in Pruszków, Pruszków, 05-800 Poland; 5 Department of Propaedeutics, Physical Diagnostics and Dental Physiotherapy, Research Club STO-MATER-FIZ, Pomeranian Medical University in Szczecin, Szczecin, 71-252 Poland; 6 DENTAL CENTER WEJT & TAWAKOL in Szczecin, Szczecin, 70-546 Poland; 7 Department of Medical Equipment, Pomeranian Medical University in Szczecin, Szczecin, 70-204 Poland

**Keywords:** Temporomandibular disorders, Dental students, Psychological stress, Prevalence, Systematic review, DC/TMD, RDC/TMD

## Abstract

**Background:**

Temporomandibular disorders (TMD) are a group of musculoskeletal conditions that may affect quality of life and functional performance. Dental students represent a population potentially at increased risk due to academic demands, psychosocial stress, and occupational strain; however, reported prevalence estimates and associated factors vary across studies.

**Objective:**

This systematic review aimed to synthesize evidence published between 2010 and 2024 on the prevalence of TMD among dental students and to examine reported associations with psychological stress, gender, and stage of dental education.

**Methods:**

A systematic literature search was conducted in PubMed, Scopus, Web of Science, and Embase in accordance with PRISMA 2020 guidelines. The search strategy combined controlled vocabulary (MeSH and Emtree terms) and free-text terms. Original quantitative studies involving dental students were included. Data extraction included study characteristics, diagnostic methods, prevalence estimates (n/N, %), and reported associations. Methodological quality was assessed using an adapted Newcastle–Ottawa Scale, and the certainty of evidence was evaluated using the GRADE framework. Due to substantial heterogeneity in diagnostic criteria and outcome measures, a qualitative synthesis was performed, with studies grouped according to diagnostic approach. Missing data were coded as “NA”, and non-reportable prevalence values were indicated as “NR”.

**Results:**

A total of 23 studies were included. Reported prevalence of TMD ranged from 15.0% to 82.3%, reflecting differences in diagnostic methods and study designs. Studies using standardized diagnostic criteria (RDC/TMD or DC/TMD) generally reported lower prevalence estimates compared to those using self-reported instruments. Psychological stress was assessed in 15 out of 23 studies; however, reported associations with TMD were not consistent. Associations with gender were also inconsistently reported. The overall certainty of evidence was rated as low.

**Conclusions:**

Temporomandibular disorders are frequently reported among dental students; however, prevalence estimates vary considerably due to methodological heterogeneity. Reported associations with psychological stress and gender are inconsistent, and the predominantly cross-sectional design of included studies precludes causal inference. Future research should prioritize standardized diagnostic criteria, validated assessment tools, and longitudinal designs to improve comparability and strengthen the evidence base.

**Supplementary Information:**

The online version contains supplementary material available at 10.1186/s12903-026-08719-3.

## Introduction

Temporomandibular disorders (TMD) comprise a heterogeneous group of musculoskeletal and psychosomatic conditions affecting the temporomandibular joints, masticatory muscles, and associated structures. Patients with TMD commonly present with pain, joint sounds, restricted mandibular movement, and functional impairment, which may substantially reduce quality of life and psychological well-being [1–3]. Due to the complex anatomy and function of the temporomandibular joint (TMJ), which coordinates mastication, speech, and swallowing, dysfunction within this system can have wide-ranging clinical and psychosocial consequences [4–6]. TMD are widely recognized as multifactorial in origin, resulting from interactions between biological, behavioral, and psychosocial factors [7, 8]. Proposed contributors include occlusal disturbances, trauma, parafunctional habits such as bruxism or clenching, postural overload, and psychological stress [9–11]. Psychological distress may influence pain perception, muscle activity, and coping behaviors, thereby increasing vulnerability to TMD symptoms [12–15]. Epidemiological data suggest that clinically relevant TMD affect approximately 5–12% of the general population, while signs or symptoms such as joint sounds or transient pain may occur in up to 70% of individuals during their lifetime [16–18]. The condition most frequently affects young adults, with a higher reported prevalence among women, although gender-related findings remain inconsistent across studies [19, 20]. Dental students represent a population of particular interest in TMD research. Dental education is characterized by high academic demands, prolonged static postures, fine motor precision, and continuous performance pressure, all of which may contribute to both physical and psychological strain [21–23]. Multiple studies have shown that dental students experience elevated stress levels compared with students in other academic disciplines, particularly during clinical training and examination periods [24–26]. Such stress may promote parafunctional habits, increased muscle tension, and maladaptive postures, thereby increasing the risk of developing or exacerbating TMD symptoms [27–29]. Previous reviews have identified an association between psychological stress and TMD in student populations; however, many were limited by narrow inclusion criteria, small sample sizes, or heterogeneous diagnostic approaches [30–32]. Moreover, studies have employed diverse diagnostic instruments ranging from standardized clinical criteria (RDC/TMD, DC/TMD) to self-reported questionnaires, leading to wide variability in reported prevalence estimates. In addition, most available studies are cross-sectional, limiting causal inference and hindering the development of evidence-based preventive strategies. In recent years, changes in academic environments, including increased digital workload, remote learning components, and evolving educational structures, have renewed interest in the psychosocial and musculoskeletal health of healthcare students [33, 34]. Elevated levels of stress, anxiety, and physical strain associated with these changes have been reported among university students, particularly within demanding healthcare curricula. These factors further underscore the need for an updated and comprehensive synthesis of the literature focusing specifically on dental students. Therefore, the aim of this systematic review was to synthesize evidence published between 2010 and 2024 regarding the prevalence of temporomandibular disorders among dental students and to examine associated risk factors, with particular emphasis on psychological stress. By summarizing findings across different countries, diagnostic criteria, and assessment tools, this review seeks to identify consistent patterns, methodological limitations, and key gaps in the literature, thereby informing future research directions and preventive strategies within dental education.

### Aim of the study

The aim of this systematic review was to synthesize available evidence published between 2010 and 2024 on temporomandibular disorders (TMD) among dental students. Specifically, the review sought to summarize reported prevalence estimates of TMD and to descriptively examine associated factors, including psychological stress, gender, and stage of dental education. A further objective was to explore how differences in diagnostic criteria and assessment tools used across studies may contribute to variability in reported prevalence and associated findings. By providing a structured qualitative synthesis of studies conducted in diverse academic and geographic settings, this review aims to identify consistent patterns, methodological limitations, and key gaps in the literature to inform future observational and interventional research in dental education.

## Materials and methods

### Protocol and reporting standards

This systematic review was conducted in accordance with the Preferred Reporting Items for Systematic Reviews and Meta-Analyses (PRISMA 2020) guidelines. The review protocol was prospectively registered in the International Prospective Register of Systematic Reviews (PROSPERO; registration number: CRD420251141416), ensuring methodological transparency and minimizing the risk of reporting bias.

### Search strategy

A comprehensive literature search was performed in four electronic databases: PubMed, Scopus, Web of Science, and Embase. The search covered studies published between January 2010 and April 2024. The search strategy combined free-text terms and controlled vocabulary (e.g., MeSH terms in PubMed and Emtree terms in Embase). The following keywords and their synonyms were used: (“temporomandibular disorders” OR “TMD” OR “temporomandibular joint dysfunction” OR “TMJ disorders”) AND (“dental students” OR “dentistry students” OR “undergraduate dental students”) AND (“prevalence” OR “epidemiology” OR “risk factors” OR “psychological stress” OR “anxiety”) The full search strategies for each database are provided in Supplementary Material. No restrictions were applied regarding geographic location or study design during the initial search. Only studies published in English were included. Additionally, the reference lists of included studies and relevant reviews were manually screened to identify further eligible articles.

### Eligibility criteria

Studies were included if they met the following criteria:

- original quantitative studies published between 2010 and 2024;
- conducted among dental students as the primary study population;
- reported prevalence of temporomandibular disorders and/or associations with psychological or psychosocial factors;
- used standardized diagnostic criteria (e.g., RDC/TMD, DC/TMD) or validated self-report instruments (e.g., Fonseca Anamnestic Index).

Studies were excluded if they:

- included non-dental student populations without separate subgroup analysis;
- were reviews, editorials, case reports, conference abstracts, or letters;
- lacked sufficient quantitative data to extract prevalence or associations;
- were not published in English.

### Study selection

Two reviewers independently screened titles and abstracts for eligibility. Full-text articles were then assessed for inclusion. Discrepancies were resolved through discussion or consultation with a third reviewer. The study selection process is presented in the PRISMA 2020 flow diagram. All reports identified for full-text review were successfully retrieved (reports not retrieved = 0).

### Data extraction

Data were independently extracted by two reviewers using a standardized extraction form. The following variables were collected:

- author and year of publication;
- country of study;
- study design;
- sample size;
- diagnostic criteria or assessment tools for TMD;
- reported prevalence of TMD (n/N, %);
- methods used to assess psychological stress or psychosocial variables;
- reported associations with gender and academic stage.

When prevalence values were not explicitly reported but could be derived from available data, they were calculated. Studies that did not provide sufficient information to determine a standardized prevalence were marked as “NR” (not reported).

### Assessment of methodological quality and risk of bias

The methodological quality of included studies was assessed using an adapted version of the Newcastle–Ottawa Scale (NOS) for cross-sectional studies.

The scale evaluates three domains:

1. Selection (representativeness of the sample, sample size, non-respondents);
2. Comparability (control for confounding factors);
3. Outcome (assessment method and statistical analysis).

Each study was assigned a score ranging from 0 to 9:

- 0–3: low quality.
- 4–6: moderate quality.
- 7–9: high quality.

Quality assessment was performed independently by two reviewers. Disagreements were resolved through discussion. Inter-rater agreement was not formally quantified, which is acknowledged as a limitation. Detailed item-level assessments are provided in Supplementary Material.

## Certainty of evidence (GRADE Assessment)

The overall certainty of evidence for key outcomes (TMD prevalence, association with psychological stress, and gender differences) was evaluated using the GRADE (Grading of Recommendations Assessment, Development and Evaluation) framework. Evidence from observational studies was initially rated as low certainty and could be downgraded or upgraded based on the following factors:

- risk of bias;
- inconsistency of results;
- indirectness;
- imprecision;
- publication bias.

A summary of the GRADE assessment is presented in Table 2.

### Data synthesis

Due to substantial heterogeneity in study design, diagnostic criteria (RDC/TMD, DC/TMD, self-reported tools), and outcome measures, a quantitative meta-analysis was not performed. Instead, a structured qualitative synthesis was conducted. Studies were grouped according to diagnostic approach to improve interpretability of prevalence estimates. Associations between TMD and selected variables (gender and psychological stress) were summarized descriptively and visualized using a binary heatmap. Not all studies assessed both variables; therefore, missing evaluations were coded as “NA”.

## Results

### Study selection

**Fig. 1:**
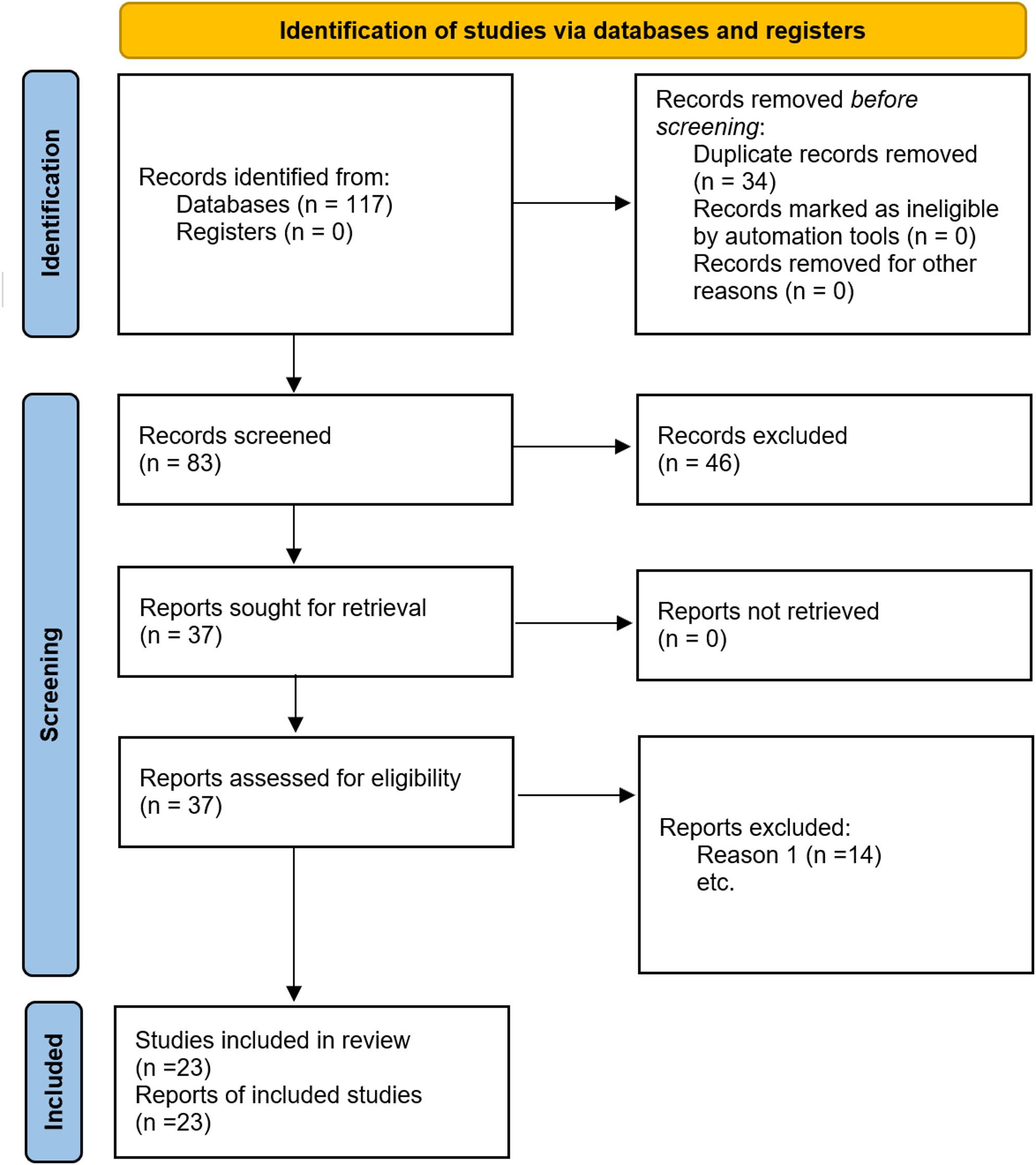
PRISMA 2020 flow diagram illustrating the study selection process for the systematic review

The database search identified a total of 117 records. After removal of 34 duplicates, 83 records remained for title and abstract screening. Of these, 46 were excluded. A total of 37 full-text articles were assessed for eligibility, all of which were successfully retrieved (reports not retrieved = 0). Fourteen studies were excluded due to not meeting the inclusion criteria (e.g., non-dental student populations, insufficient quantitative data, or non-English language). Ultimately, 23 studies were included in the qualitative synthesis. The detailed study selection process is presented in Fig. 1 (PRISMA flow diagram). No automation tools were used during the study selection process; all records were independently screened manually by two reviewers.

### General characteristics of included studies

The characteristics of the included studies are summarized in Table 1. A total of 23 studies published between 2010 and 2023 were included, representing diverse geographic regions. Sample sizes varied considerably across studies, reflecting heterogeneity in study design and population characteristics. The included studies employed different approaches to assess temporomandibular disorders (TMD), including standardized diagnostic criteria such as RDC/TMD and DC/TMD, as well as validated self-report instruments (e.g., Fonseca Anamnestic Index). For clarity, studies were grouped according to diagnostic methodology in Table 1. Across the included studies, the reported prevalence of TMD ranged from 15.0% to 82.3%, depending on the diagnostic method and study population. Studies using standardized diagnostic criteria (RDC/TMD or DC/TMD) reported prevalence estimates ranging approximately from 15.0% to 66.9%, whereas studies based on self-reported instruments reported higher and more variable estimates, ranging from approximately 35.0% to 82.3%. For studies that reported sufficient data, prevalence values were extracted or calculated as n/N (%) and are presented in Table 1. Overall, the findings indicate that TMD is reported among dental students; however, prevalence estimates should be interpreted cautiously due to substantial methodological heterogeneity across studies.

**Table 1:** Characteristics of included studies on temporomandibular disorders (TMD) among dental students (2010–2024), including country, study design, diagnostic methods, prevalence (n/N, %), and reported associations with gender and psychological stress

No.	Author (Year)	Country	Study design	Diagnostic method	Prevalence (*n*/*N*, %)	Gender association	Stress association
1.	Marklund et al. (2010) [31]	Sweden	Longitudinal cohort	RDC/TMD	84/280 (30.0%)	+	-
2.	Rocha et al. (2017) [35]	Brazil	Cross-sectional	Fonseca Index	52/90 (57.8%)	-	+
3.	Bicaj et al. (2017) [36]	Kosovo	Cross-sectional	RDC/TMD	90/166 (54.0%)	-	-
4.	Rani et al. (2017) [29]	India	Cross-sectional	RDC/TMD	84/580 (15.0%)	-	-
5.	Azevedo et al. (2018) [37]	Brazil	Cross-sectional	RDC/TMD	38/105 (36.2%)	-	-
6.	Lövgren et al. (2018) [38]	Sweden	Cross-sectional	RDC/TMD	54/157 (30.0%)	+	-
7.	Câmara-Souza et al. (2018) [34]	Brazil	Cross-sectional	Fonseca Index	28/80 (35.0%)	-	-
8.	Lung et al. (2018) [39]	Australia	Cross-sectional	Questionnaire-based	105/136 (77.2%)	+	-
9.	Jivnani et al. (2019) [40]	India	Cross-sectional	RDC/TMD	34/200 (17.0%)	-	+
10.	Ortiz-Culca et al. (2019) [41]	Peru	Cross-sectional	DC/TMD	497/2562 (19.4%)	+	-
11.	Alahmary et al. (2019) [42]	Saudi Arabia	Cross-sectional	Fonseca Index	52/105 (49.5%)	+	-
12.	Alkhudhair et al. (2018) [43]	Saudi Arabia	Cross-sectional	Questionnaire-based	NR (self-reported symptoms only; *n* = 226)	NA	+
13.	Owczarek et al. (2020) [44]	Poland	Cross-sectional	RDC/TMD	NR (*n* = 200)	NA	+
14.	Namvar et al. (2021) [45]	Iran	Cross-sectional	RDC/TMD	60/120 (50.0%)	+	+
15.	Kirarslan et al. (2021) [33]	Turkey	Cross-sectional	RDC/TMD	91/246 (37.0%)	-	-
16.	Srivastava et al. (2021) [46]	India	Cross-sectional	RDC/TMD	91/246 (37.0%)	+	+
17.	Somoskövi et al. (2023) [47]	Hungary	Cross-sectional	DC/TMD	25–56/102 (24.5–54.9%) depending on diagnostic criteria	-	+
18.	Benassi et al. (2022) [48]	Italy	Cross-sectional	DC/TMD	35/90 (38.9%)	-	+
19.	Homeida et al. (2022) [49]	Sudan	Cross-sectional	Fonseca Index	119/240 (49.5%)	+	+
20.	Eraslan et al. (2022) [30]	Turkey	Cross-sectional	RDC/TMD	303/452 (66.8%)	+	-
21.	Ahuja et al. (2018) [50]	India	Cross-sectional	Fonseca Index	370/450 (82.3%)	-	+
22.	Nazir et al. (2023) [51]	Saudi Arabia	Cross-sectional	DC/TMD	216/323 (66.9%)	-	-
23.	Faulin et al. (2015) [52]	Brazil	Cross-sectional	RDC/TMD	46/126 (36.5%)	-	-

Abbreviations:

*RDC/TMD* – Research Diagnostic Criteria for Temporomandibular Disorders;

*DC/TMD* – Diagnostic Criteria for Temporomandibular Disorders;

*NR* – not reported;

*NA* – not assessed;

“+” – statistically significant association;

“–” – no statistically significant association;

### Association with psychological stress and gender

**Fig. 2:**
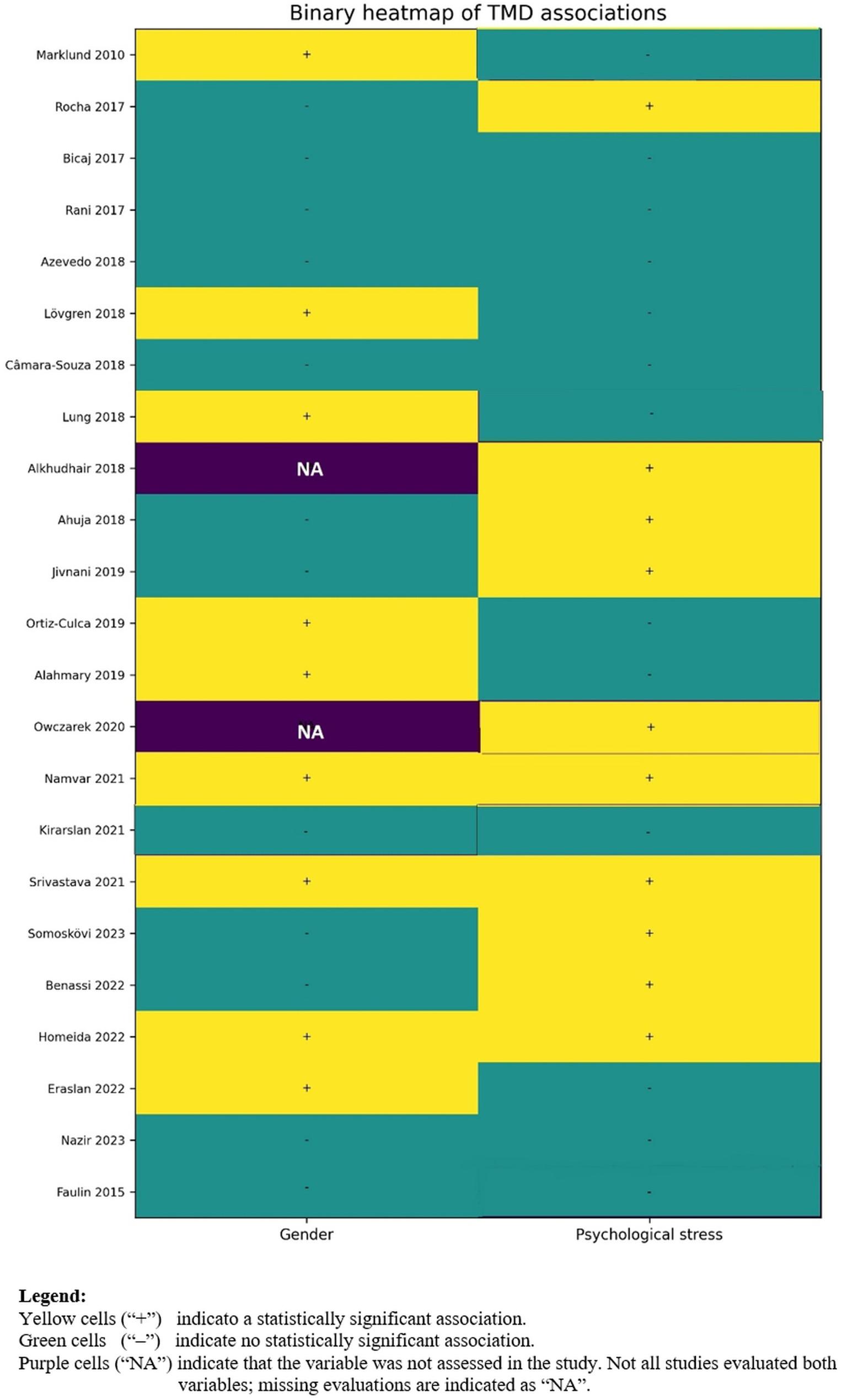
Binary heatmap showing reported associations between temporomandibular disorders (TMD), gender, and psychological stress across the included studies

The association between TMD and selected variables (psychological stress and gender) is summarized in Fig. 2. Not all included studies evaluated both variables; therefore, studies that did not assess a given variable are indicated as “NA” in the heatmap. Psychological stress was assessed in 15 out of 23 studies, whereas gender was evaluated in 21 out of 23 studies. A total of 10 out of 23 studies reported a statistically significant association between psychological stress and TMD; however, findings were not consistent across all studies. For gender, a statistically significant association with TMD was reported in 9 out of 21 studies that assessed this variable, while two studies did not evaluate gender (NA). Overall, these findings demonstrate variability in reported associations across studies. Given the predominance of cross-sectional study designs, these associations should be interpreted as correlational rather than causal.

### Risk of bias and methodological quality

The methodological quality of the included studies, assessed using the Newcastle–Ottawa Scale (NOS), is summarized in Supplementary Table S1. Most studies were of moderate to high methodological quality. Common limitations included lack of sample size justification and insufficient reporting of non-respondents. Detailed item-level assessments are provided in the Supplementary Material.

### Certainty of Evidence

The certainty of evidence for the main outcomes (TMD prevalence, association with psychological stress, and gender differences) was assessed using the GRADE (Grading of Recommendations Assessment, Development and Evaluation) framework. All included studies were observational and predominantly cross-sectional in design; therefore, the initial level of evidence was rated as low. The overall certainty of evidence for all assessed outcomes (TMD prevalence, association with psychological stress, and gender differences) was rated as low according to the GRADE framework. This reflects the observational nature of the included studies, methodological heterogeneity, and variability in outcome reporting. The certainty of evidence was further evaluated across five domains: risk of bias, inconsistency, indirectness, imprecision, and publication bias. Downgrading of evidence certainty was applied due to methodological limitations, heterogeneity in diagnostic criteria, and variability in outcome reporting across studies. A summary of the GRADE assessment is presented in Table 2.

**Table 2:** Summary of findings and certainty of evidence (GRADE)

Outcome	No. of studies	Study design	Risk of bias	Inconsistency	Indirectness	Imprecision	Publication bias	Overall certainty
TMD prevalence	23	Cross-sectional	Serious	Serious	Not serious	Serious	Suspected	Low
TMD & stress	15	Cross-sectional	Serious	Serious	Not serious	Serious	Suspected	Low
TMD & gender	18	Cross-sectional	Serious	Moderate	Not serious	Serious	Suspected	Low

## Discussion

### Principal findings

This systematic review provides an overview of the prevalence of temporomandibular disorders (TMD) among dental students and their associations with selected demographic and psychosocial factors. The findings indicate that TMD is a relatively common condition in this population, although prevalence estimates varied considerably across studies. This variability may be related to differences in diagnostic approaches, study populations, and methodological quality. Studies using standardized diagnostic criteria such as RDC/TMD or DC/TMD generally reported more conservative prevalence estimates compared to those based on self-reported instruments, highlighting the importance of methodological consistency when interpreting epidemiological data. This observation is consistent with previous reports emphasizing the variability of TMD prevalence depending on diagnostic methodology [35, 36, 50].

### Interpretation of associations

A number of included studies reported statistically significant associations between TMD and psychological stress, as well as gender. These findings are in line with the biopsychosocial model, which proposes that both biological and psychosocial factors may contribute to TMD [45, 51]. However, it is important to emphasize that the majority of included studies were cross-sectional in design. Therefore, the observed associations should be interpreted as correlational rather than causal. Similar limitations have been highlighted in previous studies examining TMD and psychosocial factors in student populations [37, 47]. Similarly, although higher prevalence of TMD was frequently observed among female students, this finding should be interpreted cautiously, as potential confounders (e.g., hormonal, behavioral, or psychosocial variables) were not consistently controlled across studies. Previous literature also suggests that gender differences in TMD may reflect complex interactions between biological and psychosocial factors rather than a single underlying mechanism [44, 53].

### Methodological considerations

The included studies demonstrated substantial heterogeneity in diagnostic criteria, assessment tools, and outcome reporting, which influenced both prevalence estimates and reported associations and limited the possibility of conducting a quantitative meta-analysis, necessitating a qualitative synthesis [54, 55]. Importantly, not all studies assessed the same variables. As reflected in Fig. 2, some studies did not evaluate psychological stress or gender, which may influence the overall interpretation of associations. Another relevant consideration is the variability in diagnostic approaches. While RDC/TMD and DC/TMD provide standardized clinical criteria, self-reported instruments may be more susceptible to reporting bias and overestimation of prevalence. This limitation has been discussed in previous epidemiological studies on TMD [54, 55].

### Risk of bias and quality of evidence

The methodological quality of included studies was generally moderate to high, as assessed using the Newcastle–Ottawa Scale. However, several limitations were identified, including inadequate reporting of sample size justification and non-response rates. Additionally, inter-rater agreement for quality assessment was not formally quantified, which may limit reproducibility. This limitation has been acknowledged in similar systematic reviews of observational studies [56]. Importantly, the overall certainty of evidence for all outcomes was rated as low, which limits the confidence in the observed associations and prevalence estimates. Therefore, the findings should be interpreted with caution, particularly when considering potential clinical or educational implications. This reflects the observational nature of the included studies, heterogeneity in methodology, and variability in outcome reporting.

### Clinical and educational implications

The findings indicate that TMD is reported among dental students; however, the clinical significance and underlying mechanisms remain uncertain. Some studies reported associations with psychological stress, although these findings should be interpreted cautiously. Educational environments characterized by high academic demands may contribute to increased stress levels. Similar observations have been reported in studies evaluating stress-related disorders among healthcare students [47, 51]. Therefore, preventive strategies such as stress management programs, early screening, and increased awareness of TMD symptoms could be considered in dental education settings.

### Limitations of the review

This review has several limitations that should be considered when interpreting the findings. First, only studies published in English were included, which may introduce language bias and limit the generalizability of the results. Second, substantial heterogeneity was observed across the included studies in terms of diagnostic criteria (RDC/TMD, DC/TMD, and self-reported instruments), assessment tools, study populations, and outcome reporting. This variability limited direct comparability between studies and precluded the performance of a quantitative meta-analysis [54, 55]. Third, not all studies assessed the same variables, particularly psychological stress and gender. Missing evaluations were coded as “NA”, and in some cases prevalence data were reported as “NR” due to insufficient information. Although this approach improves transparency, it may reduce the completeness and comparability of the findings. Fourth, the majority of included studies were cross-sectional in design, which limits the ability to establish temporal relationships and precludes causal inference between TMD and associated factors. Fifth, variability in diagnostic methods may have introduced measurement bias. Studies based on self-reported instruments may overestimate prevalence compared to those using standardized clinical criteria [54, 55]. Sixth, although the methodological quality of included studies was generally moderate to high, several limitations were identified, including insufficient reporting of sample size justification, response rates, and control of confounding factors. Additionally, inter-rater agreement in the quality assessment process was not formally quantified, which is consistent with limitations reported in similar reviews of observational studies [56]. Seventh, the possibility of publication bias cannot be excluded, as studies reporting statistically significant findings may be more likely to be published. Finally, the overall certainty of evidence, assessed using the GRADE framework, was rated as low [28]. This reflects limitations in study design, inconsistency of findings, and imprecision in reported estimates, and limits confidence in the observed prevalence estimates and reported associations; therefore, the findings should be interpreted with caution.

### Future directions

Future research should aim to use standardized diagnostic criteria such as DC/TMD to improve comparability across studies. Longitudinal designs are needed to better understand the temporal relationships between TMD and psychosocial factors. Additionally, more comprehensive reporting of methodological details, including sample size justification, response rates, and use of validated instruments, would enhance the quality of evidence in this field, as recommended in previous methodological studies [56].

## Conclusion

Within the limitations of this review, temporomandibular disorders are reported among dental students; however, prevalence estimates vary considerably across studies (15.0%–82.3%) due to substantial methodological heterogeneity. Reported associations with psychological stress and gender are inconsistent, and the predominantly cross-sectional design of included studies precludes causal inference. The overall certainty of evidence was rated as low, which limits confidence in the observed findings. Future research should prioritize the use of standardized diagnostic criteria, validated assessment tools, and longitudinal study designs to improve comparability and strengthen the evidence base.

## Supplementary Information

Supplementary Material 1.

## Data Availability

Availability of Data and Materials: The datasets supporting the conclusions of this article are included within the article and its supplementary materials. No new datasets were generated. Additional data extraction sheets and quality assessment details are available from the corresponding author upon reasonable request.

## Abbreviations

DC/TMD: Diagnostic Criteria for Temporomandibular Disorders
GRADE: Grading of Recommendations Assessment, Development and Evaluation
NA: Not assessed
NOS: Newcastle–Ottawa Scale
NR: Not reported
PRISMA: Preferred Reporting Items for Systematic Reviews and Meta-Analyses
PROSPERO: International Prospective Register of Systematic Reviews
RDC/TMD: Research Diagnostic Criteria for Temporomandibular Disorders
TMJ: Temporomandibular joint
TMD: Temporomandibular disorders
