# Supplementary material for: Temporomandibular joint disorders in dental students - a systematic review of the literature
Source: BMC Oral Health. 2026 May 30;26:1490. doi: 10.1186/s12903-026-08719-3 (PMC13483644; doi:10.1186/s12903-026-08719-3)
Supplement: Supplementary file 1 [file 12903_2026_8719_MOESM1_ESM.docx]

**Supplementary Material**

**Detailed Search Strategies**

The following search strategies were developed for each database using a combination of free text terms and controlled vocabulary (where applicable). Searches were adapted to the syntax and indexing system of each database.

**1. PubMed (MEDLINE)**

("Temporomandibular Joint Disorders"
OR "temporomandibular disorder*"
OR "TMD"
OR "TMJ disorder*"
OR "temporomandibular joint dysfunction")
AND
("Students, Dental"
OR "dental student"
OR "dentistry student*"
OR "undergraduate dental student")
AND
("Prevalence"
OR "epidemiology"
OR "risk factor"
OR "associated factor*"
OR "psychological stress"
OR "stress"
OR "anxiety")

**Filters applied:**

- Publication date: January 2010 – April 2024
- Language: English

**2. Scopus**

(TITLE-ABS-KEY ("temporomandibular disorder"
OR "TMD"
OR "TMJ disorder"
OR "temporomandibular joint dysfunction"))
AND
(TITLE-ABS-KEY ("dental student"
OR "dentistry student*"
OR "undergraduate dental student"))
AND
(TITLE-ABS-KEY ("prevalence"
OR "epidemiology"
OR "risk factor"
OR "associated factor"
OR "psychological stress"
OR "stress"
OR "anxiety"))

**Limits applied:**

- Year: 2010–2024
- Language: English

**3. Web of Science Core Collection**

TS= ("temporomandibular disorder"
OR "TMD"
OR "TMJ disorder*"
OR "temporomandibular joint dysfunction")
AND
TS= ("dental student"
OR "dentistry student"
OR "undergraduate dental student")
AND
TS= ("prevalence"
OR "epidemiology"
OR "risk factor"
OR "associated factor"
OR "psychological stress"
OR "stress"
OR "anxiety")

**Filters applied:**

- Timespan: 2010–2024
- Language: English

**4. Embase**

('temporomandibular joint disorder'
OR 'temporomandibular disorder'
OR 'TMD'
OR 'TMJ disorder'
OR 'temporomandibular joint dysfunction')
AND
('dental student'
OR 'dental student'
OR 'dentistry student'
OR 'undergraduate dental student')
AND
('prevalence'
OR 'epidemiology'
OR 'risk factor'
OR 'psychological stress'
OR 'anxiety'
OR 'stress')

**Limits applied:**

- Publication years: 2010–2024
- Language: English

| **Study** | **Representativeness** | **Sample**  **size** | **Non-**  **respondents** | **TMD**  **assessment** | **Comparability** | **Outcome**  **assessment** | **Statistics** | **Reporting** | **Total** |
| --- | --- | --- | --- | --- | --- | --- | --- | --- | --- |
| Marklund 2010 | * | * | – | * | * | * | * | * | 8 |
| Rocha 2017 | * | – | – | * | * | * | * | * | 7 |
| Bicaj 2017 | * | – | – | * | – | * | * | * | 6 |
| Rani 2017 | * | * | – | * | – | * | * | * | 7 |
| Azevedo 2018 | * | – | – | * | – | * | * | * | 6 |
| Lövgren 2018 | * | – | – | * | * | * | * | * | 7 |
| Câmara-Souza 2018 | * | – | – | * | – | * | * | * | 6 |
| Lung 2018 | * | – | – | – | * | * | * | * | 6 |
| Alkhudhair 2018 | * | – | – | – | * | * | * | * | 6 |
| Ahuja 2018 | * | * | – | * | * | * | * | * | 8 |
| Jivnani 2019 | * | – | – | * | * | * | * | * | 7 |
| Ortiz-Culca 2019 | * | * | – | * | * | * | * | * | 8 |
| Alahmary 2019 | * | – | – | * | * | * | * | * | 7 |
| Owczarek 2020 | * | – | – | * | * | * | * | * | 7 |
| Namvar 2021 | * | – | – | * | * | * | * | * | 7 |
| Kirarslan 2021 | * | * | – | * | * | * | * | * | 8 |
| Srivastava 2021 | * | – | – | * | * | * | * | * | 7 |
| Somoskövi 2023 | * | – | – | * | * | * | * | * | 7 |
| Benassi 2022 | * | – | – | * | * | * | * | * | 7 |
| Homeida 2022 | * | – | – | * | * | * | * | * | 7 |
| Eraslan 2022 | * | * | – | * | * | * | * | * | 8 |
| Nazir 2023 | * | * | – | * | * | * | * | * | 8 |
| Faulin 2015 | * | – | – | * | – | * | * | * | 6 |

**Supplementary Material**

**Table S1. Detailed item-level assessment of methodological quality using the Newcastle–Ottawa Scale (NOS)**

The methodological quality of included studies was assessed using an adapted Newcastle–Ottawa

Scale (NOS) for cross-sectional studies. Each study was evaluated across three domains: Selection, Comparability, and Outcome. Stars (*) indicate fulfillment of a given criterion.

**NOS Domains and Criteria**

**Selection (max 4 stars):**

1. Representativeness of the sample
2. Sample size justification
3. Non-respondents
4. Ascertainment of exposure (diagnostic method for TMD)

**Comparability (max 2 stars):**
5. Control for confounding factors (e.g., age, gender, stress)

**Outcome (max 3 stars):**
6. Assessment of outcome (validated tool / clinical criteria)
7. Statistical test appropriateness
8. Reporting of results (confidence intervals, p-values)

**Table S1**

**Legend**

* = criterion fulfilled

– = criterion not fulfilled or not reported

**Interpretation of total score:**

- **0–3** → low quality
- **4–6** → moderate quality
- **7–9** → high quality
